# Neurotechnological solutions for post‐traumatic stress disorder: A perspective review and concept proposal

**DOI:** 10.1049/htl2.12055

**Published:** 2023-11-28

**Authors:** Richard Laugharne, Mohsen Farid, Christopher James, Anirban Dutta, Christopher Mould, Noelle Molten, Jonathan Laugharne, Rohit Shankar

**Affiliations:** ^1^ Psychoanalytica Community Interest Company St German UK; ^2^ Cornwall Intellectual Disability Equitable Research University of Plymouth and Cornwall Partnership NHS Foundation Trust Truro UK; ^3^ Data Science Research Centre University of Derby Derby UK; ^4^ School of Engineering University of Warwick Warwick UK; ^5^ Biomedical Engineering Department University of Lincoln Lincoln UK; ^6^ House of Opportunity Salisbury UK; ^7^ Tech4Dev Ogudu, Lagos Nigeria; ^8^ PAX Centre Perth Australia

**Keywords:** biomedical communication, feedback, health care, learning (artificial intelligence), patient care, patient rehabilitation, patient treatment, risk analysis

## Abstract

Post‐traumatic stress disorder (PTSD) is an anxiety condition caused by exposure to severe trauma. It is characterised by nightmares, flashbacks, hyper‐vigilance and avoidance behaviour. These all lead to impaired functioning reducing quality of life. PTSD affects 2–5% of the population globally. Most sufferers cannot access effective treatment, leading to impaired psychological functioning reducing quality of life. Eye movement desensitisation and reprocessing (EMDR) is a non‐invasive brain stimulation treatment that has shown significant clinical effectiveness in PTSD. Another treatment modality, that is, trauma‐focused cognitive behavioural therapy is also an effective intervention. However, both evidence‐based treatments are significantly resource intensive as they need trained therapists to deliver them. A concept of a neuro‐digital tool for development is proposed to put to clinical practice of delivering EMDR to improve availability, efficiency and effectiveness of treatment. The evidence in using new technologies to measure sleep, geolocation and conversational analysis of social media to report objective outcome measures is explored. If achieved, this can be fed back to users with data anonymously collated to evaluate and improve the tool. Coproduction would be at the heart of product development so that the tool is acceptable and accessible to people with the condition.

## 1 INTRODUCTION

Post‐traumatic stress disorder (PTSD) is an anxiety disorder precipitated by severe trauma and characterised by the symptoms of nightmares, flashbacks, hypervigilance, avoidance of reminders of the trauma [1]. This is alongside associated negative thoughts and feelings and an exaggerated startle response [1]. These symptoms are persistent for at least a few weeks and impair functioning [1]. The prevalence of PTSD is between 2% and 5% of the population in World Health Organisation Surveys [2].

There are effective psychological therapies for PTSD, notably trauma focused cognitive behavioural therapy and eye movement desensitisation and reprocessing (EMDR), both recommended by the National Institute of Clinical Excellence (NICE) in the United Kingdom [3]. EMDR, which is a non‐invasive brain stimulation treatment, has been found to be superior to other therapies in reducing PTSD scores in meta‐analyses [4, 5]. However, these therapies are labour intensive and expensive when delivered by trained clinicians.

In low‐middle income countries (LMIC) mental health resources can be limited especially due to relatively few trained health professionals [2]. However, the needs can be high, and PTSD common. In LMIC 76–85% of people with severe mental illness receive no treatment, and 75% of people with PTSD do not even seek treatment, possibly because they know little is available [2].

Self‐administered EMDR therapy may be made available via the digital delivery of a smartphone application at a very low cost with little or no clinical supervision required. Likewise, trauma‐focused CBT can be made available via smart phone application [6]. The US Department of Veterans Affairs has developed an app called PTSD Coach [7], which has been evaluated in several countries [8, 9, 10, 11, 12]. Bilateral stimulation apps have been developed but have not been evaluated sufficiently yet [13]. This project seeks to help people with PTSD in situations with very limited mental health resources receive therapy through digital means and improve the quality of their lives.

This study aims to develop:

- A digital tool to help people suffering from PTSD access effective, personalised, holistic help with a smartphone or computer when clinical services are not accessible.
- A digital tool they can use with a friend, family member or treatment partner and can create a virtual peer‐support community.
- A tool that uses smartphone and wearables data to unobtrusively monitor progress‐ data such as sleep monitoring, social media activity, geolocation and conversational analysis [14]—as well as conventional symptom scores and subjective feedback.
- A tool that uses machine learning and AI algorithms to assess the trauma the subject presents with and provide clinically sound advice. It will also spot instances of possible harm the subject may experience.

## 2 METHODS PROPOSED

The development of the tool would encompass the three key areas of developing and evidencing the tool capability, build a logical concept and ensure these are underpinned by co‐production with experts by experience.

### 2.1 Tool capabilities

We want the tool to have the following properties:

1. Psychoeducation: The tool will describe PTSD to users in a clearly understandable way and describe how this condition affects people and can be treated.
2. Diagnostic assessment: It is important to help people to understand the difference between temporary distress caused by trauma which naturally resolves itself and longer standing PTSD symptoms that may require treatment.
3. Symptom rating scale: If they do have PTSD, a symptom rating scale quantifies how much the condition is affecting them and gives a baseline to the level of distress suffered.
4. Choice of treatments and treatment delivery: There are two non‐pharmaceutical therapies recommended by NICE: eye movement desensitisation and reprocessing (EMDR) and trauma focused cognitive behavioural therapy (T‐F CBT). We aim to offer both treatments via the smartphone app intelligent enough to give users a choice of therapy. We will be able to measure the effectiveness of these therapies in different groups and situations.
5. Subjective measure of user satisfaction of the app: The app will ask users to evaluate the app and its effectiveness in achieving its goals.
6. Repeating the symptom rating scale monthly: This can be provided as easy to understand visualisation for the user to assess if the treatment is helping.
7. Behavioural and other types of data will be collected: The application will inform users of the need to collect behavioural, biometric, social (via social media) and other social activities such as geolocations. Users will be informed of the data that need to be collected and the app, which will be GDPR aware, and will request their approval to share the data.
8. Feedback to client: The collected and computed data will be shared with the user in a variety of formats using cutting edge user experience technologies (UX) to keep them informed of their progress. The app will also provide frequently ask questions (FAQs) and will allow the user to make and receive answers to their questions when possible.
9. Building an intelligent knowledge base: As data is being collected, the app will build a language model and data warehouse that provide clinical insights about the types of traumas, user types, age, geographical locations, and other details, such as a user eco‐system. Such data will be used to build intelligence in the system and will help the clinical understanding of PTSD and other types of traumas which consequently could potential help to enhance treatment and other support modalities.
10. Evidence based: The tool will be quality assured through the NICE Digital Framework and seek to achieve the Organisation for the Review of Care and Health Apps (ORCHA) approval.

### 2.2 Concept development

#### 2.2.1 Literature, product and technology reviews

It is essential we have a good grounding on the evidence base of what works for people suffering with PTSD in the digital space. There are established literature reviews that underpin the NICE clinical guidelines for treating PTSD [3] but the digital treatments are early in their development. A greater understanding of the neurophysiological underpinnings of EMDR may help us to develop better digital interventions. We will conduct rapid literature reviews (registered on PROSPERO) in key areas for this product, including:

1. Neurophysiological understanding of EMDR effects: The neurophysiological understanding of EMDR can improve the delivery of the therapy. The review can generate testable hypotheses on how EMDR works to integrate traumatic memory into general semantic networks. Repetitive redirection of attention in EMDR can activate the brain's REM sleep system, leading to the activation of specific areas of the anterior cortex of the cingulate gyrus, facilitating its function as a filter. EMDR integration reduces traumatic memory strength and PTSD negative affect via hippocampal and amygdaloid pathways. EMDR may activate the lateral cerebellum, leading to thalamic and prefrontal cortex involvement in memory integration. Posterior cerebellum and metabolic connectivity with the precuneus impact EMDR's effectiveness in PTSD treatment. Neurophysiological testing and brain imaging can help study the cerebellocortical circuit for EMDR's mechanisms.
2. Biomarkers and PTSD: We will examine how digital sleep measurements correlate with EEG monitoring of sleep and relate to PTSD treatment response. We will examine the evidence for using geolocation and social‐media conversational analysis as treatment outcomes in PTSD.
3. The effectiveness and safety of digitally delivered treatments for PTSD: There are established digital tools delivering trauma focused CBT and at least six bilateral movement tools which are earlier in their development.
4. PTSD diagnostic and symptom rating scales: There is good evidence established in this area.
5. Other areas: We will also conduct product and technology reviews of digital tools for PTSD looking at digital delivery for trauma‐focused cognitive behavioural therapies and bilateral eye movement tools.

### 2.3 Coproduction

Co‐production is the process of enabling the experiences of people and organizations to contribute to the care and support of people. Digital applications, as products, need to reflect the general concept of co‐production for the enablement, support and welfare and wellbeing of others. Therefore, implementing co‐production as an essential foundation for any digital solution aiming to help people is paramount. It is essential that the design of the project involves people with lived experience of PTSD from the beginning and incorporates their insights on how the digital application may be of value to its users. Coproduction will need to be integral to the design, the prototype, product implementation and development, product testing and eventually product roll‐out. We will create a narrative of the proposed system which engages user forums.

It will be important to understand the lived experience of patients with PTSD from different backgrounds, that is, etiology, demographic and socioeconomic. Traumas leading to PTSD include serious accidents, physical or sexual assault, abuse, traumatic events at work, certain health problems such as being admitted to intensive care, childbirth experiences, such as losing a baby, war, conflict and torture. We will seek one to three representatives from each of these groups to contribute to the design of the product.

We will seek to understand with our co‐production partners:

1. People's experiences of PTSD, coping and engaging with health care systems and community assets.
2. Optimise any intervention built using the digital tool and its cultural content to address diversity of the PTSD groups.
3. Understand the human story reinforcing positive identities/bonding social capital whilst bringing diverse viewpoints.
4. We will follow the medical research council principles of developing complex interventions, involving supporting co‐production partners to identify the potential mental health benefits and develop a virtual cultural experience to improve PTSD symptoms.
5. Investigate potential mechanisms of mental health benefits of the digital tool we produce.
6. We will collect feedback (e.g. emotional activation, aesthetic engagement, interaction, social and cognitive stimulation, physical activity) from our co‐production groups.
7. We will focus on the lessons learnt to improve the tool performance as we publish new editions of the tool.

## 3 PRESENTING RESULTS AND FUTURE PLANS

Results and future plans would look to measuring conventional outcomes of the tool and explore assistive technology, including technological markers for routine daily health and social activity. Alongside this product development and feasibility testing will be undertaken.

### 3.1 Measuring conventional outcomes

We will measure the effectiveness of the tool in three ways:

1. Patient‐focused subjective feedback: We will put the person at the centre of the analysis and ask them if they feel the tool has helped them with their problems. We will examine if their subjective experience is correlated with the other outcomes.
2. Validated diagnostic rating scales: We will use validated symptom rating scales and ask users to complete at regular time intervals. We can chart these scores to both feedback progress to the user and inform the tool of its effectiveness.
3. Machine learning, AI and natural language processing: With the users’ consent, we will use machine learning, AI and natural language processing tools to examine biometrics such as sleep, geolocation and actimetry to examine activity, and conversational data, that is, textual analysis on social media use. We can feedback progress to the user and assess if these outcomes correlate with subjective and conventional measures.

The tool will collect a variety of anonymous data to examine patterns of effectiveness for different interventions, using biometric, demographic, socioeconomic and social interaction data. The data will be curated and fed into machine learning algorithms that will learn implied behaviours in the data and create models that will be used in the treatment of subjects based on their particular state. This will help inform continued development of the tool as well as in gaining insights into PTSD itself.

### 3.2 Biometrics for sleep: A risk matrix for the underpinning technology

We will examine the technology we want to utilise to provide innovative biofeedback and data analytics. We will examine pulse oximetry and electrodermal activity (through smart‐watches or smart‐rings), voice and video recordings and ballistocardiography. We will assess these for technical feasibility, intellectual property (IP), regulation and for the business models to use.

### 3.3 Actimetry and geolocation for social activity

PTSD often leads to social withdrawal and people staying at home where they feel safe. In using actimetry and geolocation we can measure correlates of behaviour and tell if people are able to go out more, this could contribute to an overall measure of their social functioning.

### 3.4 Language analysis for social media text

We will use natural language processing utilising the following techniques:

1. Sentiment analysis: The detection of positive and negative emotions, strength of emotion and sudden shifts in sentiment.
2. Topic modelling: To identify clusters of related topics.
3. Named entity recognition to extract specific entities such as people, locations and events to identify patterns in events.
4. Time series analysis: To analyse the temporal pattern of text data such as the frequency of certain topics or words over time. This can help to detect sudden spikes or drops in interest or activity.
5. Network analysis: To build networks or graphs of related entities such as people, locations and events based on the co‐occurrence of these entities in text.

### 3.5 Product development for the proof of concept

We will need to develop a protocol for the proof of concept with a view to building a team and securing grants/investment for software engineering and integration, and also design a product that is acceptable and inviting for users with good human computer interaction (HCI). Initially this will be a device which is initially UK based but we will be seeking an international applicability.

### 3.6 Feasibility

The pilot product will need to be tested initially for feasibility and this may need to be delivered in the United Kingdom where clinicians are present to enable a good evaluation. However, we will be always aiming to develop a product which can be used where clinicians are scarce and the device can be used by users alone or with a treatment friend who is not necessarily an expert. We also aim to create an on‐line capable community through the device which may develop into a peer support community.

## 4 DISCUSSION

There are range of emerging neuropsychiatric technologies which are patient facing and have shown to have various levels of effectiveness in user empowerment across a range of conditions from epilepsy to neurodevelopmental disorders [15, 16, 17, 18, 19, 20, 21]. The strengths of our review and proposal include the vision of a low‐cost treatment for people who have no access to clinicians to help them with their PTSD. This digital tool could help a lot of people and is scalable. It could democratise healthcare—getting help would not depend on having money to pay for it and access to expert clinicians, although the client would need to have a smartphone and for maximum benefit other wearables. If the client does have the smartphone, it would be a low resource and low maintenance solution. The exciting possibility is that in using a smartphone, enhanced with the addition of a smart‐watch or smart‐ring data, we can expand the measurement of the effectiveness of the intervention. With machine learning and AI technology the data collected can give the user and the research team real time personalised evaluations which can increase the effectiveness of the intervention. We can use big data to evaluate and feedback to the client how they are functioning.

The potential limitations include the need for users to trust our product and this needs to be built through the coproduction principle. If people are to use it, they need to trust us and the product and trust that their data is being used in their interests and for the public good. There is the challenge of translation to other languages. New translation technologies may need to be utilised. Developing the tool in commonly used languages will make the tool widely available, but we can explore if translation tools may make the tool more universal. There will be fear of risk, that the tool may make some people feel worse or even suicidal [15, 22]. Our literature reviews and feasibility trials will need to cover these risks. We will need to use good implementation science and good marketing in order to ensure the tool is acceptable and accessible to people. Some professional associations may be reticent in approving digital tools and we will approach them to understand their concerns and address them.

We believe this is a great opportunity to leap into a new age of helping many people who could not access help in the past and utilise new technologies in AI, machine learning and big data. Fundamentally the limits of availability of therapists can be overcome which will democratise healthcare and create new capable communities in helping the poor and the sick.

## AUTHOR CONTRIBUTIONS

**Richard Laugharne**: Conceptualization; data curation; formal analysis; funding acquisition; investigation; methodology; validation; visualization; writing—original draft. **Mohsen Farid**: Conceptualization; investigation; methodology; visualization; writing—review and editing. **Christopher James**: Conceptualization; formal analysis; funding acquisition; investigation; project administration; supervision; validation; visualization; writing—review and editing. **Anirban Dutta**: Conceptualization; investigation; methodology; visualization; writing—review and editing. **Christopher Mould**: Conceptualization; project administration; writing—review and editing. **Noelle Molten**: Conceptualization; project administration; visualization; writing—review and editing. **Jonathan Laugharne**: Conceptualization; methodology; validation; visualization; writing—review and editing. **Rohit Shankar**: Conceptualization; data curation; formal analysis; funding acquisition; investigation; methodology; project administration; resources; supervision; validation; visualization; writing—review and editing.

## CONFLICT OF INTEREST STATEMENT

Richard Laugharne and Rohit Shankar are directors of Psychoanalytica, which is a non‐commercial social enterprise Psychoanalytica. Christopher James and Rohit Shankar are co‐investigators of the N‐Code EPSRC grant which funded the study group involved in designing this paper. Christopher James is the current editor‐in chief of the journal. Rohit Shankar has received institutional and research support from LivaNova, UCB, Eisai, Veriton Pharma, Neuraxpharm, Bial, Angelini, UnEEG and Jazz/GW Pharma outside the submitted work. No other author has any declared conflict of interest related to this paper.

## Data Availability

All data used for the paper is within the manuscript.
